# Supplementary material for: Simultaneous evaluation of losartan and amlodipine besylate using second-derivative synchronous spectrofluorimetric technique and liquid chromatography with time-programmed fluorimetric detection
Source: R Soc Open Sci. 2019 Apr 17;6(4):190310. doi: 10.1098/rsos.190310 (PMC6502389; doi:10.1098/rsos.190310)
Supplement: S1: Effect of different types of organized media on AML (2µg / mL) and LOS (2µg / mL). S2: Effect of diluting solvents on AML (2µg / mL) and LOS (2µg / mL). S3: Effect of pH on AML (2µg / mL) and LOS (2µg / mL). [file rsos190310supp1.docx]

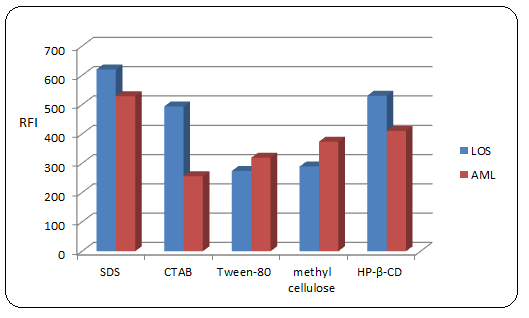


S1: Effect of different types of organized media on AML (2µg / mL) and LOS (2µg / mL)


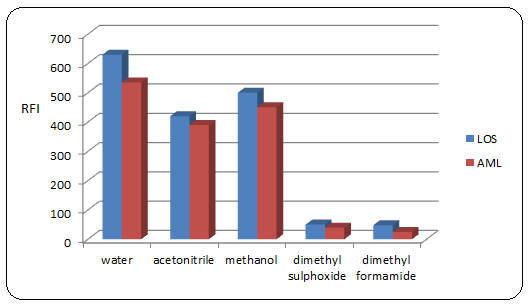


S2: Effect of diluting solvents on AML (2µg / mL) and LOS (2µg / mL)


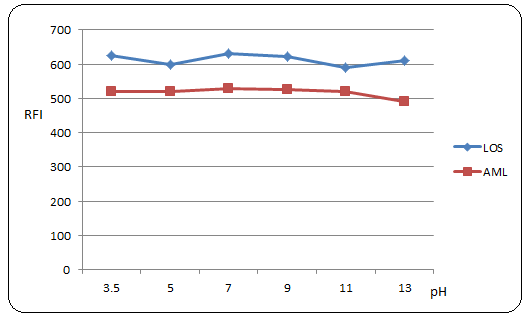


S3: Effect of pH on AML (2µg / mL) and LOS (2µg / mL).
